# Sustainable Intervention: Grape Pomace Flour Ameliorates Fasting Glucose and Mitigates Streptozotocin-Induced Pancreatic Damage in a Type 2 Diabetes Animal Model

**DOI:** 10.3390/ph17111530

**Published:** 2024-11-14

**Authors:** Raphaela Cassol Piccoli, William Sanabria Simões, Solange Vega Custódio, Kelen Cristiane Machado Goularte, Karina Pereira Luduvico, Julia Eisenhardt de Mello, Anita Avila de Souza, Ana Carolina Teixeira, Diego Araujo da Costa, Alethéa Gatto Barschak, Bruna Ferrary Deniz, Wellington de Almeida, Paula Pereira, Marisa Nicolai, Roselia Maria Spanevello, Francieli Moro Stefanello, Rejane Giacomelli Tavares, Maria Lídia Palma

**Affiliations:** 1 Postgraduation Program in Biochemistry and Bioprospection, Federal University of Pelotas, Campus Capão do Leão, S/N, Pelotas 96010-900, RS, Brazil; raphaelacassol@gmail.com (R.C.P.); williamsimoest@gmail.com (W.S.S.); solangevegacustodio@gmail.com (S.V.C.); kelenqf@gmail.com (K.C.M.G.); karina_luduvico@outlook.com (K.P.L.); julia_eisenhardt@hotmail.com (J.E.d.M.); anita_a_avila@hotmail.com (A.A.d.S.); anacarolinateixeira_@live.com (A.C.T.); 2 Postgraduation Program in Nutrition and Foods, Federal University of Pelotas, Campus Universitário, S/N, Pelotas 96010-610, RS, Brazil; diegoacostapel@gmail.com; 3 Clinical Analysis Laboratory, Federal University of Health Sciences of Porto Alegre, Department of Basic Health Sciences, Porto Alegre 90050-170, RS, Brazil; aletheagatto@gmail.com; 4 Department of Physiology and Pharmacology, Federal University of Pelotas, Campus Capão do Leão, S/N, Pelotas 96010-900, RS, Brazil; bruna.deniz@ufpel.edu.br (B.F.D.); almeida.w@outlook.com (W.d.A.); 5 Center for Research in Biosciences & Health Technologies (CBIOS), Universidade Lusófona, 1749-024 Lisboa, Portugal; p1204@ulusofona.pt (P.P.); marisa.nicolai@ulusofona.pt (M.N.); lidia.palma@ulusofona.pt (M.L.P.); 6 Center for Natural Resources and Environment (CERENA), Instituto Superior Técnico (IST), Universidade de Lisboa, Av. Rovisco Pais, 1049-001 Lisboa, Portugal; 7 EPCV, School of Phycology and Life Science, Department of Live Sciences, Universidade Lusófona, Campo Grande 376, 1749-024 Lisboa, Portugal; 8 Center for Chemical, Pharmaceutical and Food Science (CCQFA), Federal University of Pelotas, Campus Universitário, S/N, Pelotas 96160-000, RS, Brazil; rspanevello@gmail.com (R.M.S.); fmstefanello@gmail.com (F.M.S.)

**Keywords:** type 2 diabetes mellitus, grape pomace flour, streptozotocin, arinto, touriga nacional, functional food

## Abstract

**Background/Objectives**: Type 2 Diabetes Mellitus (T2DM) is characterized by hyperglycemia, increased risk of cardiovascular diseases, and oxidative imbalances. This study aimed to investigate the impact of dietary supplementations with ‘Arinto’ grape pomace flour (GPF) (WGPF) and ‘Touriga Nacional’ GPF (RGPF) in an animal model of T2DM. **Methods**: T2DM was induced by a high-fat diet (HFD) for 28 days and a single dose of streptozotocin (STZ) (35 mg/kg) on the 21st day. Forty adult male Wistar rats were divided into five groups: Control (CT), T2DM, T2DM + Metformin (250 mg/kg), T2DM + 10% ‘Arinto’ GPF (WGPF), and T2DM + 10% ‘Touriga Nacional’ GPF (RGPF). On the 21st day of the experimental protocol, animals were submitted to an oral glucose tolerance test. An oral glucose tolerance test, oxidative stress parameters, biochemical analysis, and pancreas histological analyses were performed. **Results**: T2DM impaired glucose tolerance, elevated serum triglycerides and cholesterol, increased oxidative damage in the liver, and induced pancreatic histological abnormalities. However, supplementation with WGPF and RGPF demonstrated positive effects, mitigating glycemic and lipid disruptions, ameliorating oxidative stress, and protecting pancreatic Islets β-cells. **Conclusions**: Our findings highlight the protective effects of WGPF and RGPF in the adverse impacts of T2DM. Additionally, our study emphasizes the innovative use of grape pomace, a winemaking by-product, promoting sustainability by transforming waste into functional foods with significant health benefits.

## 1. Introduction

The global prevalence of Type 2 Diabetes Mellitus (T2DM), a multifactorial chronic metabolic disease, has risen significantly. According to the International Diabetes Federation Diabetes Atlas [1], more than 540 million adults aged 20 to 79 are now affected by T2DM. Key factors contributing to the disease include impaired insulin secretion due to pancreatic β-cell dysfunction and insulin resistance (IR) caused by signaling failures [2,3]. T2DM is also marked by a notable decline in glucose tolerance, accompanied by a reduction in pancreatic β-cell mass [4]. Additionally, its pathophysiology involves hyperglycemia, dyslipidemia, oxidative stress, and inflammation [2,5].

Given the wide range of factors contributing to T2DM and its association with micro- and macrovascular complications, it is critical to explore alternative treatments for its prevention and management [3]. Recent reviews have highlighted the phytochemical and pharmacological properties of natural products, particularly their antioxidant, anti-obesity, anti-inflammatory, anti-aging, and anti-hyperlipidemic effects in the context of T2DM [6,7,8,9,10,11,12,13].

Grapes (*Vitis vinifera* L., Vitaceae) are among the most widely cultivated and consumed fruit crops globally. According to the International Organization of Vine and Wine (OIV), global grape production reached approximately 85 million tons in 2019, with a significant portion dedicated to winemaking [14]. The winemaking industry, as a major agro-industrial sector, generates large quantities of waste and by-products, underscoring the need for sustainable management to reduce its environmental impact [8]. Grape pomace, the main solid organic waste produced by wineries, consists of stalks, skins, pulp, and seeds, accounting for about 25% of the total grape mass [15,16]. Although a portion of grape pomace is used as a raw material for the production of viable grape distillate (commonly known as “grappa”), as a supplement in animal feed, or as a soil fertilizer, a significant amount of this by-product is discarded annually. This disposal raises environmental concerns and represents a substantial cost to the industry [8]. Grape pomace, rich in phenolic compounds with antioxidant properties and dietary fiber, is a valuable resource that has been investigated for recycling [17,18].

Interest in transforming grape pomace into a safe, practical form is growing in response to environmental concerns and the increasing demand for natural health-promoting compounds [8]. One cost-effective and practical approach is converting grape pomace into grape pomace flour (GPF) through drying and milling processes [19,20,21]. Recent research has concentrated on the chemical composition and biotechnological potential of GPF, highlighting its anti-hyperglycemic, antioxidant, anti-inflammatory, and anti-atherosclerotic effects in human and animal models of metabolic syndrome [17,22,23,24], obesity [25], and atherosclerosis [19].

*Vitis vinifera* ‘Touriga Nacional’ are red grapes and *Vitis vinifera* ‘Arinto’ are white grapes widely cultivated across the European Union [26]. The GPF from these varieties has received positive sensory evaluations when used in baked goods [20,21,27]. These two varieties are abundant in phytochemicals, with Arinto having higher levels of extractable polyphenols, while Touriga Nacional is distinguished by its anthocyanin content [28]. These bioactive compounds are reported to modulate key pathways affected by T2DM [29]. Additionally, the flours derived from grape pomace, especially from Touriga Nacional, have significantly lower carbohydrate content, which may provide added benefits for glycemic control compared to more traditional flours [28]. Therefore, this study aimed to investigate whether GPF from the ‘Arinto’ and ‘Touriga Nacional’ grape varieties could mitigate the metabolic, biochemical, histological, and oxidative changes observed in a high-fat diet (HFD) and streptozotocin (STZ)-induced experimental model of T2DM in rats.

## 2. Results

### 2.1. Oral Glucose Tolerance Test (OGTT)

**Figure 1:**
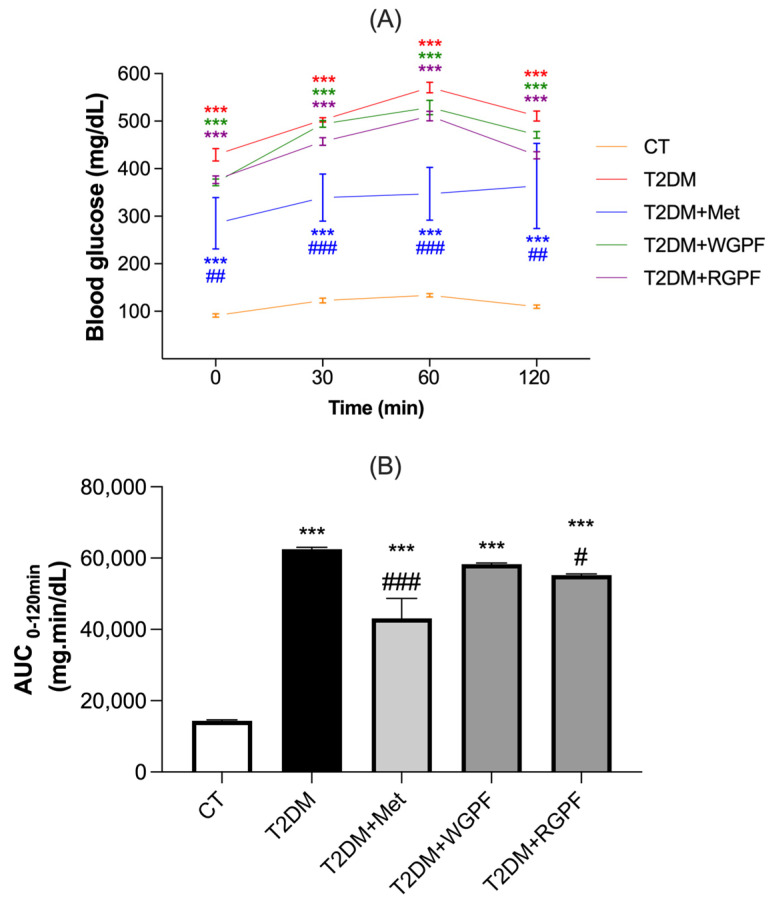
Oral glucose tolerance test demonstrating the variation in fasting blood glucose and after 30, 60, and 120 min of oral glucose overload (**A**) and the area under curve (AUC_0–120 min_) (**B**). The results are expressed as mean ± S.E.M. (n = 6–10). *** represents *p* < 0.0001 when compared to the CT group. ### represents *p* < 0.001, ## represents *p* < 0.01, and # represents *p* < 0.05 when compared to the T2DM group. Repeated-measures ANOVA followed by Bonferroni’s post hoc test. AUC, area under the curve; CT, control; T2DM, Type 2 Diabetes Mellitus; WGPF, white grape pomace flour; RGPF, red grape pomace flour; Met, metformin.

To assess glucose tolerance, all animals underwent an OGTT 72 h after the STZ injection. As illustrated in Figure 1A, the combination of an HFD and intraperitoneal STZ injections resulted in a significant increase in the baseline blood glucose levels in all diabetic groups (*p* < 0.001) compared to the CT group (95% CI 91.38–133.6). In contrast, metformin (Met) administration exhibited a protective effect by significantly reducing serum glucose levels in the T2DM + Met group (95% CI 285.2–363.7) compared to the untreated T2DM group (95% CI 429.4–570.7) (*p* < 0.01). At this point, following an oral glucose overload (2 g/kg), a significant increase in blood glucose was observed at 30, 60, and 120 min in the T2DM, T2DM + Met, white grape pomace flour (WGPF) (95% CI 371.3–528.7), and red grape pomace flour (RGPF) (95% CI 376.8–510.8) groups compared to the control group (CT) (*p *< 0.001). The interventions with GPF did not mitigate this effect. However, the group treated with Met (T2DM + Met) showed a significant reduction in glucose levels at the 30, 60, and 120 min marks compared to the untreated T2DM group (*p* < 0.001, *p* < 0.001, and *p* < 0.01, respectively). To validate the significance of the observed changes, the area under the curve (AUC) during the 120 min OGTT was calculated for all five experimental groups. As depicted in Figure 1B, the OGTT-AUC was significantly elevated in the T2DM groups compared to the CT group (*p* < 0.001, all). In contrast, treatments with Met or RGPF significantly reduced the AUC, demonstrating protection against glucose intolerance (*p* < 0.001 and *p* < 0.05, respectively).

### 2.2. Metabolic and Serum Biochemical Parameters

Table 1 presents data on feed consumption, energy intake, water intake, and weight gain over the four-week experimental period. During the pre-STZ or saline phase, the average feed consumption (in grams) was significantly lower in the T2DM + Met (*p* < 0.001) and T2DM + RGPF (*p* < 0.001) groups compared to the CT group, while the T2DM + WGPF group exhibited higher feed consumption compared to the T2DM group (*p* < 0.05). On the other hand, only the metformin-treated group showed a reduction in daily caloric intake compared to the CT and T2DM groups (*p* < 0.01 and *p* < 0.05, respectively). After the STZ or saline injection, a marked decrease in food consumption was observed in the T2DM + Met, WGPF, and RGPF groups compared to the CT group (*p* < 0.01), while no significant changes in caloric intake were observed between the experimental groups (*p* > 0.05). Regarding water intake (in milliliters), the T2DM + Met (*p* < 0.001) and T2DM + RGPF (*p* < 0.05) groups showed lower values versus the CT group. Conversely, after the STZ injection, there was a significant increase in water consumption in the T2DM (*p* < 0.001), T2DM + WGPF (*p* < 0.05), and T2DM + RGPF (*p* < 0.05) groups versus the control, whereas the T2DM + Met group showed a significant reduction in water intake versus the T2DM group (*p* < 0.05).

**Figure 2:**
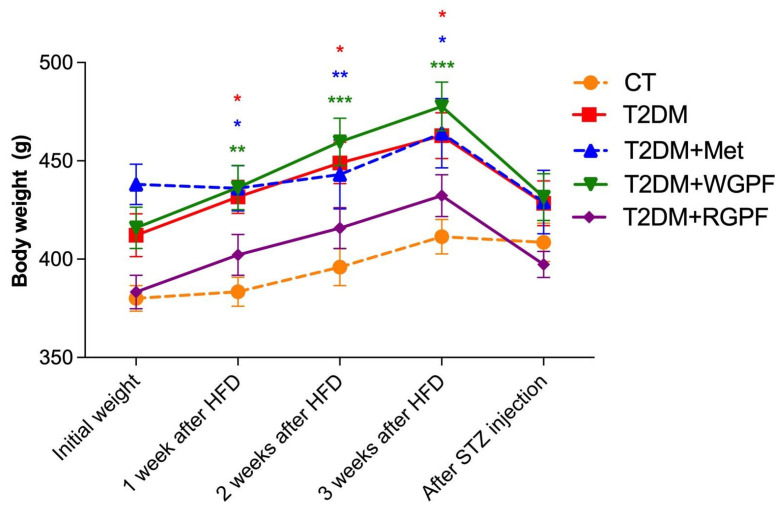
Absolute weekly body weight over three weeks of consumption of a normolipidemic diet, HFD, HFD supplemented with white grape pomace flour (WGPF), or HFD supplemented with red grape pomace flour (RGPF), and weight loss after STZ administration. Results are expressed as mean ± S.E.M. (n = 6–10). *** represents *p* < 0.001, ** represents *p* < 0.01, and * represents *p* < 0.05 versus the CT group. Repeated-measures ANOVA followed by Bonferroni’s post hoc test. CT, control; T2DM, Type 2 Diabetes Mellitus; WGPF, white grape pomace flour; RGPF, red grape pomace flour; Met, metformin.

In terms of weight gain during the pre-STZ or saline phase, the T2DM and T2DM + WGPF groups gained significantly more weight versus the CT group (*p* < 0.05). However, no significant differences were observed across the experimental groups after STZ injections (*p* > 0.05). Figure 2 illustrates the weight gain curve, where the initial weight of the T2DM + Met group (*p* < 0.01) was significantly higher than the CT group. Additionally, significantly greater body weights were observed in the T2DM, T2DM + Met, and T2DM + WGPF groups versus the CT group in the first (*p *< 0.05, *p *< 0.05, and *p *< 0.01, respectively), second (*p *< 0.05, *p* < 0.01, and *p* < 0.001, respectively), and third (*p* < 0.05, *p* < 0.05, and *p* < 0.001, respectively) weeks of the experiment. Weight loss was observed after the STZ injection in all diabetic groups, though no significant differences in total weight gain were found (*p* > 0.05). In terms of the relative pancreas weight, no significant differences were observed among the experimental groups (*p* > 0.05). However, a significant increase in visceral adipose tissue was noted in the T2DM + WGPF group versus the CT group (*p* < 0.05).

**Figure 3:**
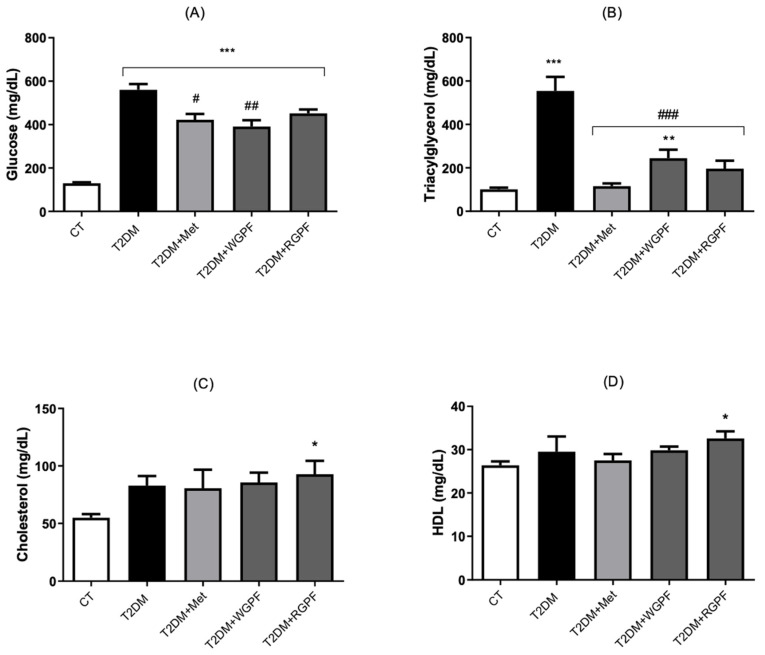
Effects of treatment with metformin and white or red grape pomace flour on serum levels of glucose (**A**), triglycerides (**B**), total cholesterol (**C**), and HDL cholesterol (**D**) in Wistar rats subjected to an experimental induction protocol of Type 2 Diabetes Mellitus. Data are presented as mean ± S.E.M. (n = 4–7). *** represents *p* < 0.001, ** represents *p* < 0.01, and * represents *p* < 0.05 versus the CT group. ### represents *p* < 0.001, ## represents *p* < 0.01, and # represents *p* < 0.05 versus the T2DM group. One-way ANOVA followed by Tukey’s post hoc test. CT, control; T2DM, Type 2 Diabetes Mellitus; WGPF, white grape pomace flour; RGPF, red grape pomace flour; HDL, high-density lipoprotein; Met, metformin.

Figure 3A–D shows the serum biochemical profile, with significant changes observed in the T2DM group versus the CT group for blood glucose (*p* < 0.001), triglycerides (TG) (*p* < 0.001), and cholesterol (*p *< 0.01). In contrast, the groups treated with Met or WGPF exhibited significantly lower blood glucose (*p* < 0.05 and *p* < 0.001, respectively) and TG (*p* < 0.001) levels versus the T2DM group. Additionally, the T2DM + RGPF group had significantly higher cholesterol levels versus the CT group (*p* < 0.01), as well as elevated high-density lipoprotein (HDL) levels (*p* < 0.05).

**Figure 4:**
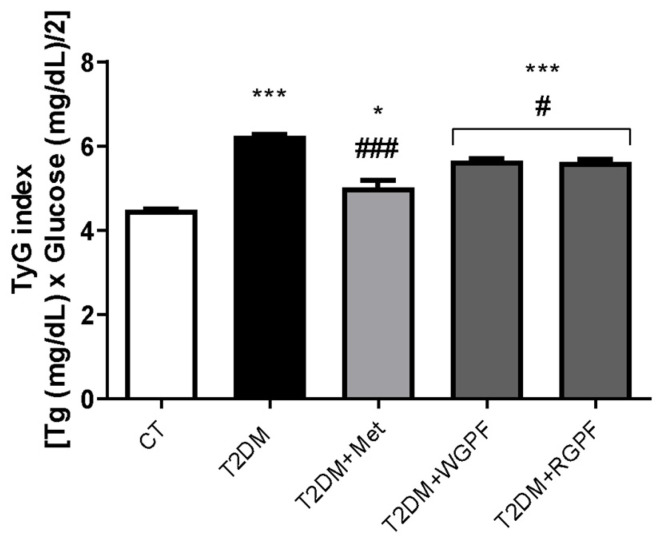
Effects of treatment with metformin and white or red grape pomace flour on the Triglyceride to Glucose Index in Wistar rats subjected to an experimental induction protocol of Type 2 Diabetes Mellitus. Data are presented as mean ± S.E.M. (n = 4–7). *** represents *p* < 0.001, and * represents *p* < 0.05 versus the CT group. ### represents *p* < 0.001, and # represents *p* < 0.05 versus the T2DM group. One-way ANOVA followed by Tukey’s post hoc test. CT, control; T2DM, Type 2 Diabetes Mellitus; WGPF, white grape pomace flour; RGPF, red grape pomace flour; Met, metformin; TG, triglycerides; TyG, triglyceride-glucose index.

The TyG index was significantly elevated in all diabetic groups (T2DM, *p *< 0.001; T2DM + Met, *p* < 0.05; T2DM + WGPF, *p* < 0.001; T2DM + RGPF, *p* < 0.001) versus the CT group. However, treatment with Met, WGPF, or RGPF significantly reduced this index versus the T2DM group (*p* < 0.001, *p* < 0.05, and *p* < 0.05, respectively) (Figure 4).

Table 2 presents data on serum markers of liver and kidney damage. Uric acid levels were significantly lower in the T2DM + Met (*p* < 0.01), T2DM + WGPF (*p* < 0.05), and T2DM + RGPF (*p* < 0.01) groups versus the CT group. Aspartate aminotransferase (AST) activity was significantly reduced in the T2DM + WGPF group versus the CT and T2DM groups (*p* < 0.01), while alanine aminotransferase (ALT) activity was significantly elevated in the T2DM group versus the CT group (*p* < 0.05). Treatments with WGPF (*p* < 0.001) and RGPF (*p* < 0.01) significantly mitigated these changes. Additionally, creatinine levels were significantly higher in the untreated T2DM group versus the CT group (*p* < 0.05). No significant differences were observed in urea or protein levels across the groups (*p* > 0.05).

### 2.3. Parameters of Oxidative Stress in the Liver

The liver oxidative stress parameters are summarized in Table 3. The coadministration of an HFD and a moderate dose of STZ significantly increased total thiol content in the T2DM, T2DM + Met, T2DM + WGPF, and T2DM + RGPF groups (*p* < 0.001) versus the CT group. However, treatment with WGPF (*p* < 0.001) and RGPF (*p* < 0.01) significantly reduced thiobarbituric acid-reactive substance (TBARS) levels, indicating lower lipid peroxidation versus the T2DM group (*p* < 0.05). No significant differences were found in reactive oxygen species (ROS) levels between the groups (*p* > 0.05). All diabetic groups showed elevated nitrite levels, and none of the treatments successfully prevented this increase (*p* < 0.001).

Regarding antioxidant enzyme activity, there were no significant differences in catalase (CAT) and superoxide dismutase (SOD) activities between the groups (*p* > 0.05). However, a significant reduction in SOD activity was observed in the T2DM (*p* < 0.001), T2DM + Met (*p* < 0.001), T2DM + WGPF (*p* < 0.001), and T2DM + RGPF (*p* < 0.01) groups versus the CT group.

### 2.4. Histopathological Analyses of the Pancreas

**Figure 5:**
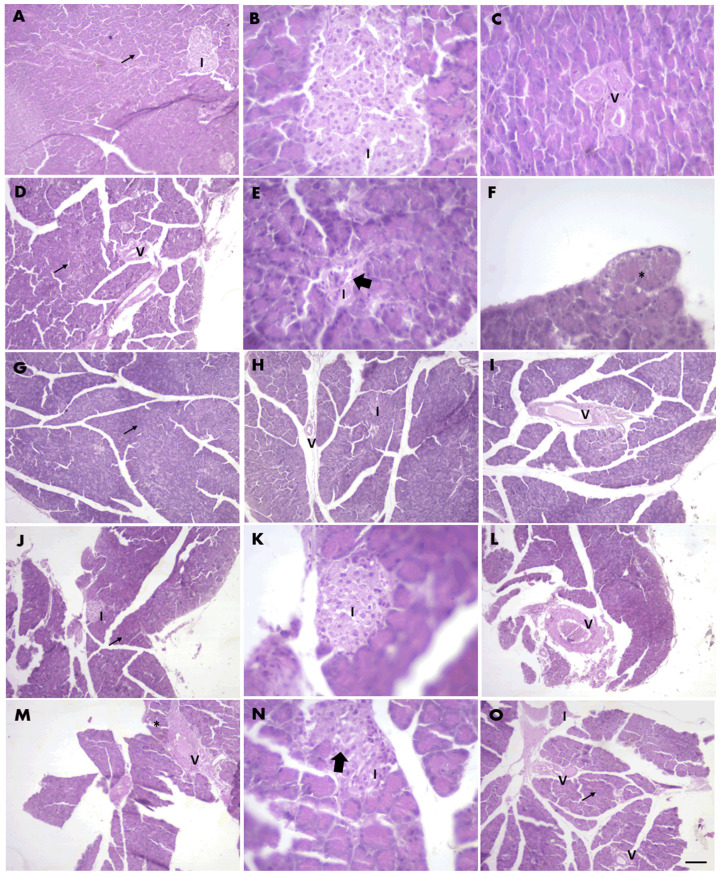
Pancreatic histological features of Wistar rats subjected to an experimental induction protocol of Type 2 Diabetes Mellitus treated with metformin or white or red grape pomace flour. (**A**–**C**) CT, control; (**D**–**F**) T2DM, Type 2 Diabetes Mellitus; (**G**–**I**) T2DM + Met, Type 2 Diabetes Mellitus + Metformin, (**J**–**L**) T2DM + WGPF, white grape pomace flour, (**M**–**O**) T2DM + RGPF, red grape pomace flour. Thin arrows, acini with normal structure; I, Langerhans islets; V, vessels; * vacuoles in acinar cells; thick arrows, spaces in islets indicating structural changes; (**D**,**I**,**L**,**M**) congested vessels. Scale: represents 100 µm in photos with 20× magnification (**A**,**C**,**D**,**F**–**J**,**L**–**O**); and represents 50 µm in photos with 40× magnification (**B**,**E**,**K**,**N**). Pancreas sections were stained with hematoxylin and eosin.

Figure 5 presents the histological analysis of the pancreas. In the CT group, the pancreatic tissue showed typical lobular organization, with intact acinar cells and well-defined islets of Langerhans, which were readily identifiable and exhibited their characteristic structure (Figure 5A–C). In contrast, the T2DM group exhibited structural alterations in the islets, characterized by increased intercellular spaces, the presence of vacuoles in some acinar cells, and vascular congestion. The overall pancreatic structure was frequently difficult to visualize, displaying disorganization in lobes and lobules. The islets of Langerhans were less discernible, both in quantity and structural integrity, with some islets showing gaps between cells. These changes, including the presence of vacuoles in acinar cells and congested vessels, are indicative of the damage caused by the experimental model (Figure 5D–F).

The pancreas in the T2DM + Met group (Figure 5G–I) displayed no significant cellular changes. This group displayed a pancreatic structure that was visually comparable to the control group, characterized by well-defined lobes and lobules, intact acinar cells, and islets that, when present, retained adequate structural characteristics. Nonetheless, minor alterations were noted, including a few congested vessels and a reduced number of identifiable islets compared to the CT group. Similarly, the T2DM + WGPF group maintained an overall intact pancreatic structure comparable to the CT group, though some swollen acinar cells with vacuoles and congested vessels were noted. While this group exhibited fewer alterations than the T2DM + RGPF group, it did not reach the level of integrity seen in the T2DM + Met and control groups. The pancreatic architecture remained largely intact, featuring identifiable lobes and lobules, although some spacing between lobules was evident. Most acinar cells and islets retained their characteristic structures, though a few swollen acinar cells, vacuoles, and congested vessels were still present (Figure 5J–L). Finally, the T2DM + RGPF group showed some congested vessels and acinar cells with swollen vacuoles. The islets of Langerhans were intact and more numerous versus the CT group, though some exhibited small spaces. The pancreatic structure was more clearly defined than in the T2DM group, although some spacing between lobes and lobules was observed. Most acini appeared intact, yet the presence of congested vessels and acinar cells with vacuoles or swelling persisted. Overall, while the islets were more intact and occurred in greater numbers, some still exhibited gaps between cells (Figure 5M–O).

## 3. Discussion

In the context of global public health, T2DM is a growing concern, with projections continually surpassing expectations in each new screening, resulting in a significant financial burden each year [1]. Natural resources, rich in bioactive compounds, have been extensively studied for their health benefits in recent years. Our research group has previously demonstrated the effects of phenolic compounds, such as anthocyanins, flavonoids, and catechins, found in these resources in animal models of T2DM [30], IR [31], and metabolic syndrome [32,33,34]. In the present study, we observed that the consumption of WGPF and RGPF significantly influenced markers of oxidative stress in the liver, lipid and glycemic profiles, and pancreatic structure in T2DM Wistar rats.

In terms of anthropometric parameters, all T2DM groups experienced a significant reduction in food intake after intraperitoneal STZ injections on day 21, resulting in notable body weight loss, aligning with previous reports in the literature [35,36]. However, these effects seemed to diminish in the days following the STZ injection. As observed by Giribabu et al. [37], grape seed extracts helped counteract weight loss in STZ-induced diabetic rats during a 28 day follow-up period.

Dysregulation of carbohydrate, lipid, and protein metabolism is central to the pathology of T2DM, leading to severe consequences, including long-term dysfunctions in patients suffering from chronic hyperglycemia [38]. Dietary polyphenols and fiber have been shown to improve lipid and glycemic profiles [39,40,41]. Consistent with our study, Rodriguez Lanzi et al. [24] reported that grape pomace administration reduced triglyceride concentrations in an animal model of metabolic syndrome induced by a high-fat, high-fructose diet. Additionally, Ferri et al. [42] demonstrated that grape pomace lowered cholesterol levels by increasing the transcriptional activity of cholesterol 7α-hydroxylase (cyp7a1), a rate-limiting enzyme in bile acid formation, crucial for maintaining cholesterol homeostasis.

The anti-hyperlipidemic effects of grape pomace may stem from a synergistic action of its bioactive compounds and dietary fiber. Soluble fiber in grape pomace can prevent lipid emulsification, affect triglyceride hydrolysis, and lower blood cholesterol, promoting cardiovascular health [28]. Accordingly, a meta-analysis available in the literature indicates that grape juice, high in simple sugars, may worsen blood glucose levels in healthy or diabetic patients, while fiber-rich grape by-products, like GPF, have beneficial effects on insulin resistance, underscoring their therapeutic potential in T2DM management [43]. Also, the antioxidant phenolic compounds in grape pomace may also reduce cholesterol absorption by disrupting micelles, decreasing cholesterol solubility and availability [19,44]. Downing et al. [45] further demonstrated that grape seed procyanidin extract reduced triacylglycerol levels while increasing the fecal excretion of cholesterol and bile acids, also decreasing hepatic lipid deposition.

In our study, the hypoglycemic effects of WGPF were evident in serum glucose levels, while RGPF showed significant effects in the OGTT test. The OGTT is recommended for assessing abnormalities in glucose homeostasis and is widely recognized as the gold standard for diagnosing diabetes. In diabetic rats, an increase in glycemia was observed, and the ability of metformin (Met) to reduce glycemia during the OGTT confirms the validity of this experimental model. Previous studies from our group have shown that dietary interventions, including the incorporation of extracts rich in bioactive compounds, can effectively reduce glucose levels and improve overall metabolic health [31,32]. Also, the literature suggests that the postprandial hypoglycemic effects of grape by-products depend on the dose, diet, and the duration of the experimental period [23,46]. Certain synthetic antidiabetic drugs work by inhibiting digestive enzymes like α-amylase and α-glucosidase to control postprandial hyperglycemia [47], and studies have shown that grape pomace may target similar mechanisms. Specific α-glucosidase inhibitors, such as 6-*O*-D-glycosides, were isolated from ‘Tinta Cão’ grape pomace [48], while phenolic compounds like catechin and anthocyanins were identified as potential α-amylase inhibitors in Merlot grape pomace via an in silico molecular docking analysis [49].

Additionally, a higher phenolic content has been associated with stronger enzymatic inhibition, and procyanidins isolated from grape seeds can lower hyperglycemia more effectively than low-dose insulin, exhibiting an insulin-mimetic effect. Campos et al. [50] investigated the effect of glycosylated anthocyanins and flavonols on glucose uptake in an in vitro cell culture model, demonstrating a reduction in glucose uptake, possibly due to the inhibition of glucose transporters SGLT1 and GLUT2. These findings highlight the potential of grape pomace in diabetes prevention and control [48,49,50].

The TyG index is a well-established marker for assessing IR, demonstrating a strong correlation with the hyperinsulinemic–euglycemic clamp technique, which is considered the gold standard for IR diagnosis [51,52]. This index not only reflects glycemic status but also serves as an indicator of cardiovascular health [52]. In patients with T2DM, elevated TyG index levels have been associated with an increased risk of cardiovascular complications [53]. In our study, Met, WGPF, and RGPF exhibited protective effects against elevated markers of the risk for cardiovascular diseases, which is consistent with the findings of De Morais et al. [54], who observed a reduction in TyG index values in rats fed a sucrose solution and treated with pterostilbene. Additionally, uric acid levels, another important marker of cardiovascular health, remained stable across the T2DM groups examined in this study.

Given the liver’s pivotal role in regulating glucose and lipid metabolism, liver dysfunction can significantly exacerbate IR and T2DM [55]. Previous studies have highlighted the hepatoprotective effects of grape-derived compounds in preventing liver damage induced by a high-fat diet [56]. Our findings align with this evidence, demonstrating that WGPF and RGPF offer protection against liver dysfunction.

In this sense, hepatic dysfunction can intensify oxidative stress, a major factor in the development of IR and T2DM-related complications [57]. In T2DM, elevated oxidative stress disrupts liver function, leading to increased triglyceride synthesis and cholesterol dysregulation. Our study demonstrated that treatment with WGPF and RGPF not only mitigated oxidative stress but also improved hepatic lipid metabolism by lowering triglyceride levels, modulating cholesterol balance, stabilizing membrane lipids, and reducing oxidative stress. These findings underscore the liver’s pivotal role in mediating the oxidative stress response and its potential as a target for therapies aimed at managing IR and T2DM.

Additionally, hyperglycemia triggers oxidative stress through various pathways [58]. Lipid peroxidation, a key marker of oxidative stress, is characterized by the interaction with ROS with polyunsaturated fatty acids. This leads to cell membrane damage, which disrupts the function of membrane-bound enzymes and receptors, contributing to diabetes progression [59]. Our study found a marked increase in lipid peroxidation, as indicated by TBARS levels, in the liver of diabetic rats, which was associated with elevated ROS levels and reduced activity of antioxidant enzymes such SOD and CAT [58]. These enzymes play a vital role in ROS defense, with SOD converting superoxide anions to hydrogen peroxide, which is then broken down by CAT [57]. Based on our findings, we propose that bioactive compounds in GPF may function as free radical scavengers and potentially activate endogenous genes encoding antioxidant enzymes such as SOD, thereby offering protection against oxidative damage in the liver of diabetic rats.

Pancreatic β-cell dysfunction plays a crucial role in the development of diabetes, with histological analyses frequently showing considerable damage to these cells in T2DM models [60]. In our study, STZ, a known β-cell cytotoxin, induced marked pancreatic alterations, including vacuole formation and structural damage, consistent with the pathological changes typically observed in T2DM [61,62]. However, treatment with Met, WGPF, and RGPF demonstrated a protective effect on pancreatic tissue. Histological evaluation confirmed reduced vacuole formation and preserved β-cell integrity, suggesting that the iron-chelating and antioxidant properties of grape pomace compounds mitigated β-cell damage. This protective mechanism aids in pancreatic function and contributes to better glucose regulation, highlighting the potential of these treatments as an adjunct in diabetes management [63].

In conclusion, our findings suggest that grape pomace flour holds significant potential in the prevention and management of T2DM by modulating oxidative stress, improving glycemic control, regulating lipid metabolism, and protecting pancreatic function.

The findings of our study demonstrate the potential protective effects of Arinto and Touriga Nacional GPF against T2DM. These effects include improvements in IR, the regulation of lipid metabolism, a reduction in oxidative damage, and the preservation of the pancreatic structure—outcomes that have not been previously documented. Our results suggest that these specific GPFs, which have not been extensively studied before, may provide protective benefits in the management of T2DM that go beyond those reported for other grape varieties. Furthermore, our study underscores the innovative use of grape pomace, a by-product of the winemaking process, contributing to sustainability by repurposing waste materials into functional foods with substantial health benefits.

## 4. Materials and Methods

### 4.1. Chemicals

Streptozotocin (STZ), dichloro-dihydro-fluorescein diacetate (DCFH-DA), epinephrine, 5,5′-dithiobis (2-nitrobenzoic acid) (DTNB) were obtained from Sigma-Aldrich Co. (St. Louis, MO, USA). The commercial kit for biochemical parameters was obtained from Bioclin (Bioclin MG—Brazil). All other reagents used in the experiments were of analytical grade and highest purity.

### 4.2. Grape Pomace Flours (GPF)

Grape pomace, a by-product of wine production, was sourced from a single production batch of red grapes (‘Touriga Nacional’) and white grapes (‘Arinto’) from Adega Cooperativa Carmim, located in the Reguengos de Monsaraz region of Alentejo, Portugal. After collection, the samples were dried using an air circulation system (J.P. Selecta, Barcelona, Spain) for 24 h at 60 °C. They were then ground using a domestic blade crusher (Moulinex, Alençon, France) to produce GPF with a particle size of ≤400 μm. The GPF was packaged in polypropylene bags, properly sealed, and stored at 4 °C ± 2 °C in a dark environment until use [20].

The phytochemical composition of both GPFs was previously characterized [28]. ‘Arinto’ GPF contains 2.9 mg of proanthocyanidins, denoted as catechin equivalents per gram of dry weight (DW), and notably lacks anthocyanins. In contrast, ‘Touriga Nacional’ GPF is rich in anthocyanins, including delphinidin-3-*O*-glucoside, petunidin-3-*O*-glucoside, and malvidin-3-*O*-glucoside. Analysis by high-performance liquid chromatography coupled with diode-array detection and electrospray ionization mass spectrometry (HPLC−DAD/ESI-MS) revealed that both GPFs are abundant in bioactive compounds, such as (+)-catechin, (-)-epicatechin, digalloylated procyanidin dimers and trimers, and a gallic acid derivative [28].

### 4.3. Diets

The HFD was prepared according to the method outlined by Oliveira et al. [61], with some modifications, and was offered ad libitum to the animals throughout the experimental duration. Diets supplemented with WGPF and RGPF included a 10% addition of these flours, as suggested by Kim et al. [64]. The flours were incorporated into the diet mixture, which was then homogenized in a food processor until a uniform blend was achieved. This mixture was molded into pellets and dried in an oven at 60 °C for 24 h. The normolipidemic control diet was the Socil^®^ brand Autoclavable Rat and Mouse Feed, which was supplied by the Central Animal Facility of the Federal University of Pelotas.

### 4.4. Animals

This study utilized forty mature male Wistar rats, aged 90 days with a mean weight of 403 g (±34 g), sourced from the Federal University of Pelotas’ Central Animal House (Pelotas, RS, Brazil). To ensure optimal welfare, the animals were (i) Housed in a controlled environment maintained at 22 °C (±1 °C); (ii) Subjected to a standardized 12 h light/dark cycle; (iii) Provided with unlimited access to water; (iv) Grouped in suitable cages with a maximum occupancy of four rats per cage. All experimental procedures involving animals adhered strictly to the National Institutes of Health’s guidelines (NIH Publications No. 8023, revised 1978). Additionally, this research received formal approval from the Institutional Ethics Committee on the Use of Animals (Approval Reference: CEUA 033578/2022-14).

### 4.5. Experimental Design

**Figure 6:**
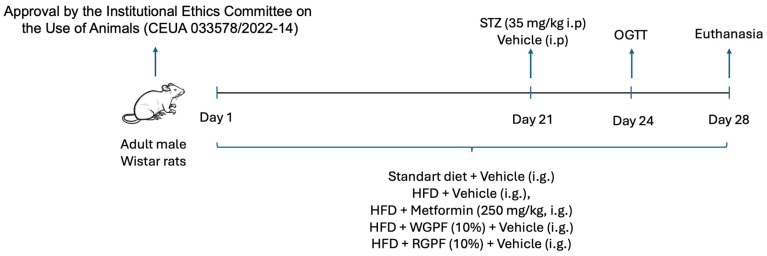
Experimental design of treatment with metformin, Arinto or Touriga Nacional grape pomace flour in an animal model of Type 2 Diabetes Mellitus. High-fat diet (HFD), metformin (Met), oral glucose tolerance test (OGTT), red grape pomace flour (RGPF), streptozotocin (STZ), white grape pomace flour (WGPF).

The experimental design is illustrated in Figure 6. Rats were randomly divided into five groups: control diet/vehicle (CT), high-fat diet/vehicle (T2DM), HFD/metformin (250 mg/kg) (T2DM + Met), HFD supplemented with 10% of WGPF (T2DM + WGPF), or a high-fat diet supplemented with 10% RGPF (T2DM + RGPF) for 4 weeks. At day 21, T2DM groups received a single intraperitoneal (i.p.) injection of STZ (Sigma-Aldrich, St. Louis, MO, USA ) (35 mg/kg) dissolved in 0.01 M sodium citrate solution [65,66]. During the trial, the animals were given a vehicle (distilled water) or Met (250 mg/kg) [67] intragastrically once a day. Body weight was recorded weekly, and food and water intake were recorded daily.

### 4.6. Sample Collection

After the 28 day experimental period and after 6 h of fasting, the animals were euthanized by deepening anesthesia with Isoflurane, followed by exsanguination through cardiac puncture and decapitation. To reduce stress, the procedure was performed in a separate room, away from the other animals. The blood was collected and centrifuged at 800× *g* for 15 min, and the resulting serum was stored at −80 °C for further biochemical assays [30]. Livers were dissected and stored for further determination of oxidative stress parameters. The visceral adipose tissue and pancreas were dissected and weighted, and the pancreas was stored for further histopathological assay.

### 4.7. Serum Biochemical Parameters

In order to investigate the metabolic disturbances related to T2DM, we measured serum glucose (mg/dL), cholesterol (mg/dL), TG (mg/dL), HDL (mg/dL), and protein (mg/dL) urea (mg/dL), uric acid (mg/dL), and creatinine (mg/dL) were measured to evaluate renal function, and ALT (U/L) and AST (U/L) activities were determined to investigate hepatic function. All biochemical parameters were tested using commercially available diagnostic kits supplied by Labtest ^®^ (Labtest, MG, Brazil). The fasting Triacylglycerol-glucose (TyG) index was calculated using the formula [Triacylglycerol (mg/dL) × Glucose (mg/dL)/2], according to Zhao et al. [68].

### 4.8. Oxidative Stress

Liver samples were homogenized in a sodium phosphate buffer, pH 7.4, containing KCl. Subsequently, the homogenates were centrifuged at 800× *g* for 10 min at 4 °C, and the resulting supernatant was utilized for the assessment of oxidative stress [69]. Protein concentration was determined by the methodologies established by Lowry et al. [70] or Bradford [71].

**Reactive oxygen species (ROS)**

Quantification of ROS production was determined by assessing the oxidation of 2′,7′-dichlorofluorescein diacetate (DCFH-DA) into fluorescent 2′,7′-dichlorofluorescein (DCF), as described by Ali et al. [72]. This parameter is reported as μmol of DCF per mg of protein.

**Thiobarbituric acid-reactive species (TBARS)**

TBARS, indicative of lipid peroxidation, were determined following the protocol delineated by Ohkawa et al. [73] and reported as nmol TBARS/mg of protein.

**Total sulfhydryl content (SH) assay**

The SH content assay was conducted through the reduction in 5,5′-dithiobis-(2-nitrobenzoate) (DTNB) by thiols, leading to the formation of oxidized disulfides (disulfide production), generating a yellow derivative (TNB), in accordance with the method outlined by Aksenov and Markesbery [74]. Results are reported as nmol of TNB per mg of protein.

**Nitrite assay**

The nitrite level was determined employing 1% sulfanilamide and 0.3% N-1-naphthylethylenediamine dihydrochloride as reagents within the Griess reaction, following the protocol detailed by Stuehr and Nathan [75]. Results are denoted as μM of nitrite per mg of protein.

**Superoxide Dismutase (SOD) activity**

This quantification of SOD activity was based on the inhibition of superoxide-dependent adrenaline auto-oxidation, as described by Misra and Fridovich [76]. The specific activity of SOD is reported as units per mg of protein.

**Catalase (CAT) activity**

CAT activity was measured by monitoring the depletion of H2O2 over a 90 s interval, according to the method of Aebi [77]. The results are reported as units per mg of protein.

### 4.9. Oral Glucose Tolerance Test (OGTT)

After 72 h of STZ administration and a 6 h fasting period, the OGTT was performed on all animals. During the fasting period, they were provided with water only and were kept in a controlled environment to which they were accustomed. The OGTT involved monitoring blood glucose levels using a glucometer (AccuChek Guide, Roche Diagnostics^®^, Indianapolis, IN, USA) at baseline (0 min) and at 30, 60, and 120 min after intragastric administration of a 50% D-glucose solution (2 mg/g). Venous blood samples were obtained at the designated time points through a small tail puncture in the animals to ensure minimally invasive handling throughout the procedure [67]. The area under the curve (AUC) for blood glucose concentrations over the time interval from 0 to 120 min was calculated using the linear trapezoidal rule method [78,79] as follows:

$$\frac{\left[C_{0}+C_{30}]\cdot [t_{30}-t_{0}\right]}{2}+\frac{\left[C_{30}+C_{60}]\cdot [t_{60}-t_{30}\right]}{2}+\frac{\left[C_{120}+C_{60}]\cdot [t_{120}-t_{60}\right]}{2}$$

where C_0_, C_30_, C_60_, and C_120_ represent blood glucose concentrations (mg/dL) measured at 0, 30, 60, and 120 min post-glucose administration, respectively, and t_0_, t_30_, t_60_, and t_120_ denote the corresponding time points (in minutes) following glucose administration.

### 4.10. Histopathology

Pancreas samples were collected, weighted fixed in 10% (pH 7.4) formaldehyde, processed by successive dehydration with ethanol baths, and embedded into paraffin blocks. Five-micrometer-thick sections (in duplicate) were cut, stained with haematoxylin-eosin (HE), and examined under a light microscope.

### 4.11. Statistical Analyses

All statistical analyses were conducted utilizing GraphPad Prism 8.0 software (GraphPad Software, San Diego, CA, USA). To assess glucose tolerance, we employed a repeated-measures ANOVA, followed by Bonferroni’s post hoc test for multiple comparisons. For normally distributed data, one-way ANOVA was applied, supplemented by Tukey’s post hoc test. Data are expressed as mean values accompanied by standard error of the mean (S.E.M.). A *p*-value of less than 0.05 (*p* < 0.05) was deemed indicative of statistically significant differences throughout the analysis.

## 5. Conclusions

**Figure 7:**
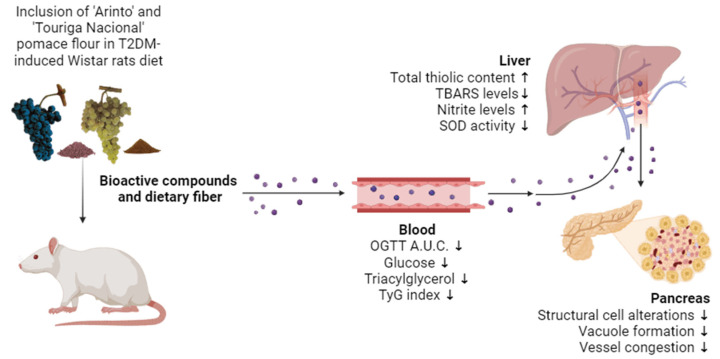
Comprehensive overview of the effects of WGPF and RGPF treatment on key parameters related to T2DM. ↑ means increase and ↓ means decrease.

The findings of our study demonstrate the potential protective effects of Arinto and Touriga Nacional GPF against T2DM (Figure 7). These effects include improvements in insulin resistance, the regulation of lipid metabolism, a reduction in oxidative damage, and the preservation of the pancreatic structure—outcomes that have not been previously documented. Our results suggest that these specific GPFs, which have not been extensively studied before, may provide protective benefits in the management of T2DM that go beyond those reported for other grape varieties. Furthermore, our study underscores the innovative use of grape pomace, a by-product of the winemaking process, contributing to sustainability by repurposing waste materials into functional foods with substantial health benefits. However, further studies are needed to better understand the specific effects of GPF on the mechanisms involved in the development and progression of T2DM, including glucose transporters and the insulin signaling pathway, including IRS-1, PI3K, AKT, and GSK-3β.

## Figures and Tables

**Table 1:** Effect of the administration of a high-fat diet and STZ, and treatment with Met and WGPF or RGPF on food consumption, energy intake, water intake, total weight gain, relative pancreas weight, and adipose tissue weight.

	CT	T2DM	T2DM + Met	T2DM + WGPF	T2DM + RGPF
**Pre STZ or saline**					
Feed consumption (g/day)	25.76 ± 0.67	21.35 ± 1.46	18.48 ± 0.92 ***	23.93 ± 1.12 #	20.36 ± 1.09 **
Energy intake (kcal/day)	91.86 ± 1.46	88.05 ± 3.31	76.64 ± 2.06 **#	98.21 ± 3.24	83.18 ± 2.83
Water intake (mL/day)	46.53 ± 2.14	41.33 ± 2.87	32.25 ± 1.43 **	39.90 ± 2.29	35.97 ± 2.51 *
Total weight gain (g)	31.44 ± 3.07	50.67 ± 6.06 *	34.80 ± 6.78	48.50 ± 4.95 *	44.44 ± 2.87
**Post STZ or saline**					
Feed consumption (g/day)	25.06 ± 0.46	20.46 ± 2.72	15.67 ± 1.63 **	16.69 ± 1.23 **	17.58 ± 1.20 **
Energy intake (kcal/day)	89.85 ± 1.65	79.55 ± 9.72	66.84 ± 5.61	68.50 ± 5.66	70.24 ± 5.36
Water intake (mL/day)	48.80 ± 1.45	107.83 ± 15.38 ***	67.3 ± 9.51 #	80.66 ± 7.04 *	83.67 ± 5.96 *
Total weight gain (g)	28.56 ± 4.38	16.25 ± 3.46	19.33 ± 2.59	19.75 ± 4.19	13.95 ± 4.99
Pancreas relative weight	0.41 ± 0.03	0.37 ± 0.02	0.31 ± 0.02	0.37 ± 0.04	0.33 ± 0.03
Adipose visceral tissue relative weight	11.05 ± 0.80	15.05 ± 1.17	15.20 ± 1.99	16.97 ± 1.91 *	13.43 ± 1.17

The data are presented as mean ± S.E.M. (n = 6–10). *** represents *p* < 0.001, ** represents *p* < 0.01, and * represents *p* < 0.05 versus the CT group. # represents *p* < 0.05 versus the T2DM group. One-way ANOVA followed by Tukey’s post hoc test. CT, control; T2DM, Type 2 Diabetes Mellitus; WGPF, white grape pomace flour; RGPF, red grape pomace flour; Met, metformin**.**

**Table 2:** Effect of the administration of a high-fat diet and STZ, and treatment with Met, WGPF, or RGPF on serum parameters of renal and hepatic damage of animals subjected to an experimental induction protocol of Type 2 Diabetes.

	CT	T2DM	T2DM + Met	T2DM + WGPF	T2DM + RGPF
Uric acid	1.25 ± 0.08	0.77 ± 0.21	0.50 ± 0.18 **	0.68 ± 0.12 *	0.50 ± 0.13 **
Urea	41.67 ± 0.88	37.33 ± 4.33	39.00 ± 3.70	33.38 ± 2.28	40.88 ± 1.46
Creatinine	0.40 ± 0.04	0.23 ± 0.03 *	0.30 ± 0.03	0.31 ± 0.02	0.29 ± 0.02
Protein	5.06 ± 0.25	5.02 ± 0.19	4.23 ± 0.22	4.22 ± 0.37	4.67 ± 0.19
AST activity	140.8 ± 10.44	149.8 ± 19.35	129.8 ± 14.67	82.14 ± 4.48 **##	132.7 ± 11.19
ALT activity	69.00 ± 7.33	106.7 ± 10.87 *	72.67 ± 7.45	51.56 ± 5.02 ###	65.50 ± 4.35 ##

The data are presented as mean ± S.E.M. (n = 4–7) ** represents *p *< 0.01, and * represents *p* < 0.05 versus the CT group. ### represents *p *< 0.001, and ## represents *p *< 0.01 versus the T2DM group. One-way ANOVA followed by Tukey’s post hoc test. ALT, alanine aminotransferase; AST, aspartate aminotransferase; CT, control; T2DM, Type 2 Diabetes Mellitus; WGPF, white grape pomace flour; RGPF, red grape pomace flour; Met, metformin. Uric acid expressed as mg/dL. Urea expressed as mg/dL. Creatinine expressed as mg/dL. Protein expressed as g/dL. AST activity expressed as U/L. ALT activity expressed as U/L.

**Table 3:** Effect of the administration of a high-fat diet and STZ, and treatment with Met, WGPF, or RGPF on oxidative stress parameters in the livers of animals subjected to an experimental induction protocol of Type 2 Diabetes.

	CT	T2DM	T2DM + Met	T2DM + WGPF	T2DM + RGPF
**Liver**					
Total thiol content	111.8 ± 3.08	137.6 ± 2.28 ***	139.0 ± 2.93 ***	139.2 ± 3.28 ***	143.9 ± 3.13 ***
TBARS levels	8.10 ± 0.66	12.13 ± 0.69 *	10.03 ± 0.81	7.02 ± 0.90 ###	7.16 ± 0.73 ##
ROS levels	35.03 ± 2.07	22.52 ± 1.31	27.02 ± 3.46	24.86 ± 1.52	28.60 ± 1.52
Nitrite levels	21.88 ± 2.88	41.50 ± 1.14 ***	37.60 ± 1.58 ***	43.26 ± 1.11 ***	37.49 ± 1.32 ***
CAT activity	43.28 ± 0.95	49.91 ± 6.18	42.45 ± 7.52	55.61 ± 3.24	41.42 ± 5.68
SOD activity	45.38 ± 1.72	27.85 ± 2.36 ***	29.16 ± 2.11 ***	22.07 ± 3.22 ***	32.88 ± 2.03 **

The data are presented as mean ± S.E.M. (n = 4–8). *** represents *p* < 0.001, ** represents *p* < 0.01, and * represents *p* < 0.05 versus the CT group. ### represents *p* < 0.001, and ## represents *p* < 0.01 versus the T2DM group. One-way ANOVA followed by Tukey’s post hoc test. Total thiol content denoted as nmol TNB/mg of protein, TBARS levels denoted as nmol TBARS/mg of protein, ROS levels denoted as μmol DCF/mg of protein, nitrite levels denoted as μM nitrites/mg of protein, CAT and SOD activity denoted as U/mg of protein. CAT, catalase; CT, control; T2DM, Type 2 Diabetes Mellitus; ROS, reactive oxygen species; WGPF, white grape pomace flour; RGPF, red grape pomace flour; Met, metformin; SOD, superoxide dismutase; TBARS, thiobarbituric acid-reactive substances.

## Data Availability

The datasets generated during the current study are available from the corresponding author on reasonable request.

## References

[1] [IDF] International Diabetes Federation (2021). Diabetes Atlas.

[2] Singh A., Kukreti R., Saso L., Kukreti S. (2022). Mechanistic Insight into Oxidative Stress-Triggered Signaling Pathways and Type 2 Diabetes. Molecules.

[3] Zhou Z., Sun B., Yu D., Zhu C. (2022). Gut Microbiota: An Important Player in Type 2 Diabetes Mellitus. Front. Cell. Infect. Microbiol..

[4] [WHO] World Health Organization (2019). Classification of Diabetes Mellitus.

[5] Banday M.Z., Sameer A.S., Nissar S. (2020). Pathophysiology of diabetes: An overview. Avicenna J. Med..

[6] Devalaraja S., Jain S., Yadav H. (2011). Exotic fruits as therapeutic complements for diabetes, obesity and metabolic syndrome. Food Res. Int..

[7] Xu L., Li Y., Dai Y., Peng J. (2018). Natural products for the treatment of type 2 diabetes mellitus: Pharmacology and mechanisms. Pharmacol. Res..

[8] Averilla J.N., Oh J., Kim H.J., Kim J.S., Kim J.S. (2019). Potential health benefits of phenolic compounds in grape processing by-products. Food Sci. Biotechnol..

[9] Gómez-Brandón M., Lores M., Insam H., Domínguez J. (2019). Strategies for recycling and valorization of grape marc. Crit. Rev. Biotechnol..

[10] Pivari F., Mingione A., Brasacchio C., Soldati L. (2019). Curcumin and Type 2 Diabetes Mellitus: Prevention and Treatment. Nutrients.

[11] Antonić B., Jančíková S., Dordević D., Tremlová B. (2020). Grape Pomace Valorization: A Systematic Review and Meta-Analysis. Foods.

[12] Blahova J., Martiniakova M., Babikova M., Kovacova V., Mondockova V., Omelka R. (2021). Pharmaceutical Drugs and Natural Therapeutic Products for the Treatment of Type 2 Diabetes Mellitus. Pharmaceuticals.

[13] Vivó-Barrachina L., Rojas-Chacón M.J., Navarro-Salazar R., Belda-Sanchis V., Pérez-Murillo J., Peiró-Puig A., Herran-González M., Pérez-Bermejo M. (2022). The Role of Natural Products on Diabetes Mellitus Treatment: A Systematic Review of Randomized Controlled Trials. Pharmaceutics.

[14] [OIV] International Organization of Vine and Wine, Report 2019. https://www.oiv.int/public/medias/6782/oiv-2019-statistical-report-on-world-vitiviniculture.pdf.

[15] Beres C., Costa G.N.S., Cabezudo I., James N.K.S., Teles A.S.C., Cruz A.P.G., Silva C.M., Tonon R.V., Cabral L.M.C., Freitas S.P. (2017). Towards integral utilization of grape pomace from winemaking process: A review. Waste Manag..

[16] Frum A., Dobrea C.M., Rus L.L., Virchea L.I., Morgovan C., Chis A.A., Arseniu A.M., Batuca A., Gligor F.G., Vicas L.G. (2022). Valorization of Grape Pomace and Berries as a New and Sustainable Dietary Supplement: Development, Characterization, and Antioxidant Activity Testing. Nutrients.

[17] Urquiaga I., D’Acuña S., Pérez D., Dicenta S., Echeverría G., Rigotti A., Leighton F. (2015). Wine grape pomace flour improves blood pressure, fasting glucose and protein damage in humans: A randomized controlled trial. Biol. Res..

[18] Niwano Y., Tada M., Tsukada M. (2017). Antimicrobial Intervention by Photoirradiation of Grape Pomace Extracts via Hydroxyl Radical Generation. Front. Phisiol..

[19] Rivera K., Salas-Pérez F., Escheverría G., Urquiaga I., Dicenta S., Pérez D., de la Cerda P., González L., Andia M.E., Uribe S. (2019). Red Wine Grape Pomace Attenuates Atherosclerosis and Myocardial Damage and Increases Survival in Association with Improved Plasma Antioxidant Activity in a Murine Model of Lethal Ischemic Heart Disease. Nutrients.

[20] Palma M.L., Nunes M.C., Rita G., Rodrigues M., Gothe S., Tavares N., Pego C., Nicolai M., Pereira P. (2020). Preliminary sensory evaluation of salty crackers with grape pomace flour. Biomed. Biopharm. Res..

[21] Palma M.L., Pêgo-Ferreira C., Nicolai M., Pereira P. (2021). Preliminary sensory evaluation of grape pomace flour sweet cookies. Biomed. Biopharm. Res..

[22] Hernández-Salinas R., Decap V., Leguina A., Cáceres P., Perez D., Urquiaga R., Velarde V. (2015). Antioxidant and anti hyperglycemic role of wine grape powder in rats fed with a high fructose diet. Biol. Res..

[23] Urquiaga I., Troncoso D., Mackenna M.J., Urzúa C., Pérez D., Dicenta S., de la Cerda P.M., Amigo L., Carreño J.C., Echeverría G. (2018). The Consumption of Beef Burgers Prepared with Wine Grape Pomace Flour Improves Fasting Glucose, Plasma Antioxidant Levels, and Oxidative Damage Markers in Humans: A Controlled Trial. Nutrients.

[24] Lanzi C.R., Perdicaro D.J., Antoniolli A., Fontana A.R., Miantello R.M., Bottini R., Prieto M.A.V. (2016). Grape pomace and grape pomace extract improve insulin signaling in high-fat-fructose fed rat-induced metabolic syndrome. Food Funct..

[25] Charradi K., Mahmoudi M., Bedhiafi T., Kadri S., Elkahoui S., Limam F., Aouani E. (2017). Dietary supplementation of grape seed and skin flour mitigates brain oxidative damage induced by a high-fat diet in rat: Gender dependency. Biomed. Pharmacother..

[26] Cabrita M.J., Freitas A.M.C.F., Laureano O., Di Stefano R. (2006). Glycosidic aroma compounds of some Portuguese grape cultivars. J. Sci. Food Agric..

[27] Marcos J., Carriço R., Souza M.J., Palma M.L., Pereira P., Nunes M.C., Nicolai M. (2023). Effect of Grape Pomace Flour in Savory Crackers: Technological, Nutritional and Sensory Properties. Foods.

[28] Pereira P., Palma M.L., Palma C., Borges C., Maurício E., Fernando A.L., Duarte M.P., Lageiro M., Fernandes A., Mateus N. (2024). Exploring the Benefits of Nutritional and Chemical Characteristics of Touriga Nacional and Arinto Varieties (*Vitis vinifera* L.). Foods.

[29] Naz R., Saqib F., Awadallah S., Wahid M., Latif M.F., Iqbal I., Mubarak M.S. (2023). Food Polyphenols and Type II Diabetes Mellitus: Pharmacology and Mechanisms. Molecules.

[30] Cardoso J.S., Teixeira F.C., de Mello J.E., de Aguiar M.S.S., Oliveira O.S., Saraiva J.T., Vizzotto M., Grecco F.B., Lencina C.L., Spanevello R.M. (2023). *Psidium cattleianum* fruit extract prevents systemic alterations in an animal model of type 2 diabetes mellitus: Comparison with metformin effects. Biomarkers.

[31] Cardoso J.S., Oliveira P.S., Bona N.P., Vasconcellos F.A., Baldissarelli J., Vizzotto M., Soares M.S.P., Ramos V.P., Spanevello R.M., Lencina C.L. (2017). Antioxidant, antihyperglycemic, and antidyslipidemic effects of Brazilian-native fruit extracts in an animal model of insulin resistance. Redox. Rep..

[32] Oliveira P.S., Gazal M., Flores N.P., Zimmer A.R., Chaves V.C., Reginatto F.H., Kaster M.P., Tavares R.G., Spanevello R.M., Lencina C.L. (2017). *Vaccinium virgatum* fruit extract as an important adjuvant in biochemical and behavioral alterations observed in animal model of metabolic syndrome. Biomed. Pharmacother..

[33] Oliveira P.S., Chaves V.C., Bona N.P., Soares M.S.P., Cardoso J.S., Vasconcellos F.A., Tavares R.G., Vizzotto M., Silva L.M.C., Grecco F.B. (2017). *Eugenia uniflora* fruit (red type) standardized extract: A potential pharmacological tool to diet-induced metabolic syndrome damage management. Biomed. Pharmacother..

[34] Oliveira P.S., Chaves V.C., Soares M.S.P., Bona N.P., Mendonça L.T., Carvalho F.B., Gutierrez J.M., Vasconcellos F.A., Vizzoto M., Vieira A. (2018). Southern Brazilian native fruit shows neurochemical, metabolic and behavioral benefits in an animal model of metabolic syndrome. Metab. Brain Dis..

[35] Correia-Santos A.M., Suzuki A., Anjos J.S., Rêgo T.S., Almeida K.C.L., Boaventura G.T. (2012). Induction of Type 2 Diabetes by low dose of streptozotocin and high-fat diet-fed in wistar rats. Medicine.

[36] Kobayashi M., Kurata T., Hamana Y., Hiramitsu M., Inoue T., Murai A., Horio F. (2017). Coffee Ingestion Suppresses Hyperglycemia in Streptozotocin-Induced Diabetic Mice. J. Nutr. Sci. Vitaminol..

[37] Giribabu N., Karim K., Kilari E.K., Kassim N.M., Salleh N. (2018). Anti-Inflammatory, Anti-Apoptotic and Pro-Proliferative Effects of *Vitis vinifera* Seed Ethanolic Extract in the Liver of Streptozotocin-Nicotinamide-Induced Diabetes in Male Rats. Can. J. Diabetes..

[38] Alfheeaid H.A., Alhowail A.A., Ahmed F., Zaki A.K.A., Alkhaldy A. (2023). Effect of Various Intermittent Fasting Protocols on Hyperglycemia-Induced Cognitive Dysfunction in Rats. Brain Sci..

[39] Amiot M.J., Riva C., Vinet A. (2016). Effects of dietary polyphenols on metabolic syndrome features in humans: A systematic review. Obes. Rev..

[40] Kim Y., Keogh J.B., Clifton P.M. (2016). Polyphenols and Glycemic Control. Nutrients.

[41] Peixoto C.M., Dias M.I., Alves M.J., Calhela R.C., Barros L., Pinho S.P., Ferreira I.C.F.R. (2018). Grape pomace as a source of phenolic compounds and diverse bioactive properties. Food Chem..

[42] Ferri M., Bin S., Vallini V., Fava F., Michelini E., Roda A., Minnucci G., Bucchi G., Tassoni A. (2016). Recovery of polyphenols from red grape pomace and assessment of their antioxidant and anti-cholesterol activities. New Biotechnol..

[43] Moodi V., Abedi S., Esmaeilpour M., Asbaghi O., Izadi F., Shirinbakhshmasoleh M., Behrouzian M., Shahriari A., Ghaedi E., Miraghajani M. (2021). The effect of grapes/grape products on glycemic response: A systematic review and meta-analysis of randomized controlled trials. Phytother. Res..

[44] Rahbar A.R., Mahmoudabadi M.M.S., Islam M.S. (2015). Comparative effects of red and white grapes on oxidative markers and lipidemic parameters in adult hypercholesterolemic humans. Food Funct..

[45] Downing L.E., Heidker R.M., Caiozzi G.C., Wong B.S., Rodriguez K., Rey F.D., Ricketts M.L. (2015). A Grape Seed Procyanidin Extract Ameliorates Fructose-Induced Hypertriglyceridemia in Rats via Enhanced Fecal Bile Acid and Cholesterol Excretion and Inhibition of Hepatic Lipogenesis. PLoS ONE.

[46] Martínez-Maqueda D., Zapatera B., Gallego-Narbón A., Vaquero M.P., Saura-Calixto F., Pérez-Jiménez J. (2018). A 6-week supplementation with grape pomace to subjects at cardiometabolic risk ameliorates insulin sensitivity, without affecting other metabolic syndrome markers. Food Funct..

[47] Cisneros-Yupanqui M., Lante A., Mihaylova D., Krastanov A.I., Rizzi C. (2023). The α-Amylase and α-Glucosidase Inhibition Capacity of Grape Pomace: A Review. Food Bioprocess Technol..

[48] Sun S., Kadough H.C., Zhu W., Zhou K. (2016). Bioactivity-guided isolation and purification of α-glucosidase inhibitor, 6-O-D-glycosides, from Tinta Cão grape pomace. J. Funct. Foods.

[49] Kato-Schwartz C.G., Corrêa R.C.G., Lima D.S., Sá-Nakanishi A.B., Gonçalves G.A., Seixas F.A.V., Haminiuk C.W.I., Barros L., Ferreira I.C.F.R., Bracht A. (2020). Potential anti-diabetic properties of Merlot grape pomace extract: An in vitro, in silico and in vivo study of α-amylase and α-glucosidase inhibition. Food Res. Int..

[50] Campos F., Peixoto A.F., Fernandes P.A.R., Coimbra M.A., Mateus N., Freitas V., Fernandes I., Fernandes A. (2021). The Antidiabetic Effect of Grape Pomace Polysaccharide-Polyphenol Complexes. Nutrients.

[51] Tao L.C., Xu J.N., Wang T.T., Hua F., Li J.J. (2022). Triglyceride-glucose index as a marker in cardiovascular diseases: Landscape and limitations. Cardiovasc. Diabetol..

[52] Selvi N.M.K., Nandhini S., Sakthivadivel V., Lokesh S., Srinivasan A.R., Sumathi S. (2021). Association of Triglyceride-Glucose Index (TyG index) with HbA1c and Insulin Resistance in Type 2 Diabetes Mellitus. Maedica.

[53] Grabež M., Škrbić R., Stojiljković M.P., Vučić V., Grujić V.R., Jakovljević V., Djuric D.M., Suručić R., Šavikin K., Bigović D. (2022). A prospective, randomized, double-blind, placebo-controlled trial of polyphenols on the outcomes of inflammatory factors and oxidative stress in patients with type 2 diabetes mellitus. Rev. Cardiovasc. Med..

[54] De Morais J.M.B., Cruz E.M.S., Rosa C.V.D., Cesário R.C., Comar J.F., Moreira C.C.L., Chuffa L.G.A., Seiva F.R.F. (2021). *Pterostilbene* influences glycemia and lipidemia and enhances antioxidant status in the liver of rats that consumed sucrose solution. Life Sci..

[55] Manilla V., Santopaolo F., Gasbarrini A., Ponziani F.R. (2023). Type 2 Diabetes Mellitus and Liver Disease: Across the Gut–Liver Axis from Fibrosis to Cancer. Nutrients.

[56] Buchner I., Medeiros N., Lacerda D.D.S., Normann C.A.B.M., Gemelli T., Rigon P., Wannmacher C.M.D., Henriques J.A.P., Dani C., Funchal C. (2014). Hepatoprotective and Antioxidant Potential of Organic and Conventional Grape Juices in Rats Fed a High-Fat-Diet. Antioxidants.

[57] Yaribeygi H., Sathyapalan T., Atkin S.L., Sahebkar A. (2020). Molecular Mechanisms Linking Oxidative Stress and Diabetes Mellitus. Oxidative Med. Cell. Longev..

[58] Newsholme P., Keane K.N., Carlessi R., Cruzati V. (2019). Oxidative stress pathways in pancreatic -cells and insulin-sensitive cells and tissues: Importance to cell metabolism, function, and dysfunction. Am. J. Physiol. Cell Physiol..

[59] Shabalala S.C., Johnson R., Basson A.K., Ziqubu K., Hlengwa N., Mthembu S.X.H., Mabhida S.E., Mazibuko-Mbeje S.E., Hanser S., Cirilli I. (2022). Detrimental Effects of Lipid Peroxidation in Type 2 Diabetes: Exploring the Neutralizing Influence of Antioxidants. Antioxidants.

[60] Deeds M.C., Anderson J.M., Armostrong A.S., Gastineau D.A., Hiddinga H.J., Jahangir A., Eberhardt N.L., Kudva Y.C. (2011). Single dose streptozotocin-induced diabetes: Considerations for study design in islet transplantation models. Lab. Anim..

[61] Duan J., Yang M., Liu Y., Xiao S., Zhang X. (2022). Curcumin protects islet beta cells from streptozotocin-induced type 2 diabetes mellitus injury via its antioxidative effects. Endokrynol. Pol..

[62] Li H., Zhang H., Wang T., Zhang L., Wang H., Lu H., Yang R., Ding Y. (2024). Grape Seed Proanthocyanidins Protect Pancreatic β Cells Against Ferroptosis via the Nrf2 Pathway in Type 2 Diabetes. Biol. Trace Elem. Res..

[63] Oliveira J.S., Silva A.A.N., Dias F.C.R., Oliveira E.L., Filho E.F.O., Soares P.C., Ferreira C.M.O., Junior V.A.S.J. (2022). Histomorphometric and oxidative evaluation of the offspring’s testis from type 2 diabetic female rats treated with metformin and pentoxifylline. Int. J. Exp. Pathol..

[64] Kim H., Bartley G.E., Arkiv T., Lipson R., Nah S.Y., Seo K., Yokoyama W. (2014). Dietary Supplementation of Chardonnay Grape Seed Flour Reduces Plasma Cholesterol Concentration, Hepatic Steatosis, and Abdominal Fat Content in High-Fat Diet-Induced Obese Hamsters. J. Agric. Food Chem..

[65] Srinivasan K., Asrat B.V.L., Kaul C.L., Ramarao P. (2005). Combination of high-fat diet-fed and low-dose streptozotocin treated rat: A model for type 2 diabetes and pharmacological screening. Pharmacol. Res..

[66] Singh S., Sharma R.K., Malhotra S., Posrhuraju R., Shandilya U.K. (2017). *Lactobacillus rhamnosus* NCDC17 ameliorates type-2 diabetes by improving gut function, oxidative stress and inflammation in high-fat-diet fed and streptozotocin treated rats. Benef. Microbes.

[67] Jiao Y., Wang X., Jiang X., Kong F., Wang S., Yan C. (2017). Antidiabetic effects of Morus alba fruit polysaccharides on high-fat-diet and streptozotocin-induced type 2 diabetes in rats. J. Ethnopharmacol..

[68] Zhao J., Fan H., Wang T., Yu B., Mao S., Wang X., Zhang W., Wang L., Zhang Y., Ren Z. (2022). TyG index is positively associated with risk of CHD and coronary atherosclerosis severity among NAFLD patients. Cardiovasc. Diabetol..

[69] Ramos V.P., da Silva P.G., Oliveira P.S., Bona N.P., Soares M.S.P., Cardoso J.S., Hoffmann J.F., Chaves F.C., Schneider A., Spanevello R.M. (2020). Hypolipidemic and anti-inflammatory properties of phenolic rich *Butia odorata* fruit extract: Potential involvement of paraoxonase activity. Biomarkers.

[70] Lowry O.H., Rosebrough N., Farr A.L., Randall R. (1951). Protein measurement with the Folin phenol reagent. J. Biol. Chem..

[71] Bradford M.M. (1976). A rapid and sensitive method for the quantitation of microgram quantities of protein utilizing the principle of protein–dye binding. Anal Biochem..

[72] Ali S.F., Lebel C.P., Bondy S.C. (1992). Reactive oxygen species formation as a biomarker of methylmercury and trimethyltin neurotoxicity. Neurotoxicology.

[73] Ohkawa H., Ohishi N., Yagi K. (1979). Assay for lipid peroxides in animal tissues by thiobarbituric acid reaction. Anal Biochem..

[74] Aksenov M.Y., Markesbery W.R. (2001). Changes in thiol content and expression of glutathione redox system genes in the hippocampus and cerebellum in Alzheimer’s disease. Neurosci. Lett..

[75] Stuehr D.J., Nathan C.F. (1989). Nitric oxide. A macrophage product responsible for cytostasis and respiratory inhibition in tumor target cells. J. Exp. Med..

[76] Misra H.P., Fridovich I. (1972). The role of superoxide anion in the autoxidation of epinephrine and a simple assay for superoxide dismutase. J. Biol. Chem..

[77] Aebi H. (1984). Catalase in vitro. Methods Enzymol..

[78] Chewchinda S., Leakaya N., Sato H., Sato V.H. (2021). Antidiabetic effects of *Maclura cochinchinensis* (Lour.) corner heartwood extract. J. Tradit. Complement. Med..

[79] Sato V.H., Chewchinda S., Goli A.S., Sato H., Nontakham J., Vongsak B. (2023). Oral Glucose Tolerance Test (OGTT) Evidence for the Postprandial Anti-Hyperglycemic Property of *Salacca zalacca* (Gaertn.) Voss Seed Extract. Molecules.

